# Spatial distribution and associated factors of health insurance coverage in Ethiopia: further analysis of Ethiopia demographic and health survey, 2016

**DOI:** 10.1186/s13690-020-00407-0

**Published:** 2020-03-16

**Authors:** Sewnet Adem Kebede, Alemneh Mekuriaw Liyew, Getayeneh Antehunegn Tesema, Chilot Desta Agegnehu, Achamyeleh Birhanu Teshale, Adugnaw Zeleke Alem, Yigizie Yeshaw

**Affiliations:** 1 Department of Epidemiology and Biostatistics, Institute of Public Health, College of Medicine and Health Sciences, University of Gondar, Gondar, Ethiopia; 2 School of Nursing, College of Medicine and Health Sciences and Comprehensive specialized hospital, University of Gondar, Gondar, Ethiopia; 3 Department of Physiology, School of Medicine, College of Medicine and Health Sciences, University of Gondar, Gondar, Ethiopia

**Keywords:** Universal health coverage, Health insurance coverage, Spatial distribution, Ethiopia

## Abstract

**Background:**

Health insurance is one of the instruments to achieve universal health coverage. However, in Ethiopia, the coverage of health insurance is very low and varies from place to place as well. Therefore, exploring the spatial distribution of health insurance is important to prioritize and design targeted intervention programs in the country.

**Methods:**

A total of 16,583 reproductive age group women (15–49 years) were included in this study. The Bernoulli model was used by applying Kulldorff methods using the SaTScan software to analyse the purely spatial clusters of health insurance coverage. ArcGIS version 10.3 was used to visualize the distribution of health insurance coverage across the country. Mixed-effect logistic regression analysis was also used to identify predictors of health insurance coverage.

**Results:**

Health insurance coverage among women aged 15–49 years had spatial variations across the country (Moran’s I: 0.115, *p* < 0.001). Health insurance coverage in Amhara (*p* < 0.001) and Tigray (*p* < 0.001) National Regional States clustered spatially. Reading newspapers at least once a week (Adjusted Odds Ratio (AOR) = 1.78, 95% CI: (1.18–2.68))), 40–44 years of age (AOR = 2.14, 95% CI: (1.37–3.35)), clerical working mothers (AOR = 4.33, 95% CI: (2.50–7.49)), mothers’ with secondary school education (AOR = 1.77; 95% CI: (1.21–2.58)), mothers’ with higher school education (AOR = 2.62; 95% CI: (1.63–4.23)), having more than 5 family members (AOR = 1.25; 95% CI: (1.01–1.55)) and richest wealth quantile (AOR = 3.43, 95% CI: (1.96–6.01)) were predictors of health insurance coverage among reproductive age group women in Ethiopia.

**Conclusion:**

Health insurance coverage was very low in Ethiopia and had spatial variations across the country. The hot spot areas with low health insurance coverage need more coherent and harmonized action such as strengthening financial protection through national health packages, sharing experience from regions which have better health insurance coverage and using mass media to increase awareness and confidence of potentials in the systems, which may encourage them to enrol.

## Background

Health insurance is one of the instruments to achieve universal health coverage, which is the major goal for health reform in many countries as well as a priority objective of world health organization (WHO) [1, 2]. Thus, health insurance guarantees that no person faces the risk of poverty when accessing health care nor give up on health services because of financial reasons [3, 4]. The world bank also indicated that health insurance is an essential part of Sustainable Development Goals (SDGs) in many countries [5].

According to 2017 World Health Organization (WHO) report, half of the world’s population cannot access needed health services, while 100 million people are pushed into extreme poverty each year because of health expenses. In addition, 800 million people spend at least 10% or more of their household budget on healthcare expenses [5, 6]. The WHO member states in 58 world health assembly agreed on the development of their health-financing systems by strengthening the role of prepayment for health care while diminishing direct payments, which were seen as one of the barriers to access to health care [7].

Ethiopia health system has three-tier structure, primary care level, secondary care level and tertiary care level. The primary care level is established on the district level and includes a primary hospital with population coverage of 100,000 people, local health centers with 25,000 people per health center and rural health posts with 5000 people per health post. The secondary and tertiary levels are comprised of general and specialized hospitals, and the coverage of each extends to larger portions of the population [8].

In Ethiopia, the health system is underfinanced and resources are very limited compared to other sub-Saharan Africa countries. Financial barriers are the major reasons for patients not seeking health services in health facilities [8, 9]. Initiation of compulsory health insurance (community-based health insurance (CBHI) for the agricultural and informal sectors, and social health insurance (SHI) for those employed in the formal sector) was one of the main components of the health care financing reform and Health Sector Transformation Plan in Ethiopia (2015/16–2019/20) to deliver towards the goals of Universal Health Coverage [10, 11].

Health insurance is important to all population groups, particularly it benefits for vulnerable groups such as mothers, newborns, and children to improve access to health care by establishing financial protection in an equitable and sustainable manner and enhance social inclusion for the majority of Ethiopian families via the health sector. With no health insurance and no financial autonomy, women were often unable to access care on their own especially in developing country like Ethiopia [12].

Age, level of education, residence, wealth status, travel cost, travel time, exposed to media and occupation of the mother were factors influencing variations in health insurance coverage in Ethiopia [13–19].

According to Ethiopian Demographic and Health Survey 2016, health insurance coverage has been geographically heterogeneous. However, spatial analyses have not been conducted to identify areas with hotspots (low health insurance coverage) among women in Ethiopia. Therefore, we aimed to identify the geographic variation of health insurance coverage and associated factors among women in Ethiopia using the latest EDHS 2016.

Identification of geographic distributions of health insurance and the impact of risk factors on health insurance coverage by area is important to prioritize and design targeted intervention programs to address universal health coverage through health insurance. In addition, understanding the spatial variation of health insurance coverage is crucial to design community-based intervention in the identified hotspot areas (low health insurance coverage) and to use limited resources effectively and also for proper mobilization, accumulation and allocation of money to cover the health needs of the people.

## Method and materials

### Study design, period and setting

Community-based cross-sectional study was conducted in Ethiopia from January 18 to June 27, 2016. Ethiopia is a country in the Horn of Africa with nine regional states (Afar, Amhara, Benishangul-Gumuz, Gambella, Harari, Oromia, Somali, Southern Nations, Nationalities, and People’s Region (SNNP) and Tigray) and two city administrations (Addis Ababa and Dire Dawa). I t has 68 zones, 817 districts, and 16,253 kebeles (smallest administrative units of a country). It has a population of 105 million. Of which, 43.47% of the population are less than 14 years [20, 21].

### Study population and sampling procedure

All women aged 15–49 years were included in the study. The 2016 EDHS sample was stratified and selected in two stages. In the first stage, a total of 645 Enumeration Areas (EAs) (202 in urban areas and 443 in rural areas) were selected with probability proportional to EA size and with independent selection in each sampling stratum. In the second stage of selection, a fixed number of 28 households per cluster were selected with an equal probability systematic selection from the newly created household listing. For this study individual data set was used and extracted the outcome and explanatory variables. Latitude and longitude coordinates were also taken from selected enumeration areas (clusters). The detailed sampling procedure was presented in the full EDHS report [20].

### Data collection procedure and variables

The study was conducted based on EDHSs data’s by accessing from the DHS program official database www.measuredhs.com, after permission was granted through online request by explaining the objective of our study. The outcome variable with important predictors were extracted from Ethiopia Demographic and Health Surveys individual data set. Data were extracted using STATA version 14.0. Women aged 15–49 years were interviewed using pre-tested woman’s standard questionnaire and several data from mothers or caretaker was obtained. Socio-economic and demographic information was also collected from women and household.

Health insurance coverage among women aged 15–49 were used as a dependent variable. The independent variables were age, level of education, residence, wealth status, occupation, marital status, family size and exposed to media. Universal health coverage defined as ensuring that all people have access to needed health services (including prevention, promotion, treatment, rehabilitation and palliation) of sufficient quality to be effective while also ensuring that the use of these services does not expose the user the financial hardship [22]. Health insurance defined as a type of insurance coverage that pays for medical and surgical expenses incurred by the insured [23].

### Data processing and analysis

Descriptive and summary statistics were done using STATA version 14 software. The analysis was done using STATA 14, ArcGIS 10.3 and SaTScan 9.6 software’s. The data was weighted using sampling weight, primary sampling unit, and strata before any statistical analysis to restore the representativeness of the survey and to tell the STATA to take into account the sampling design when calculating standard errors to get reliable statistical estimates.

In EDHS data, women within a cluster may be more similar to each other than women in the rest of the country. This violates the assumption independence of observations and equal variance across clusters. This implies that the need to consider the between-cluster variability using advanced models. Since the response variable was dichotomous, logistic regression and Generalized Linear Mixed Model (GLMM) were fitted. Model comparison was done based on Deviance Information Criteria (DIC). Mixed effect model with the lowest DIC was chosen (Table 1). Furthermore, the ICC value was 0.5 which informed us to choose GLMM over the basic model.

**Table 1:** Model comparison between logistic regression and mixed effect Logistic regression

Proposed models	AIC value	BIC value	DIC(−2xLL)
Logistic regression	5053.13	5275.28	4992
Mixed effect logistic regression	3929.72	4159.53	3868

Variables with ≤0.2 *p*-values in the bi-variable analysis were fitted in the multivariable model to measure the effect of each variable after adjusting for the effect of other variables. Adjusted Odds Ratio (AOR) with a 95% Confidence Interval (CI) and *p*-value < 0.05 in the multivariable model were declared as determinant factors of health insurance. Multi-collinearity was also checked using a variance inflation factor (VIF).

### Spatial autocorrelation analysis

Spatial autocorrelation (Global Moran’s I) statistic measure was used to evaluate whether health insurance coverage was dispersed, clustered, or randomly distributed in the study area. Moran’s I values close to−1 indicated disease/event dispersed, whereas I close to + 1 indicated disease/event clustered, and disease/event distributed randomly if I value was zero. A statistically significant Moran’s I (*p* < 0.05) led to the rejection of the null hypothesis and indicated the presence of spatial autocorrelation. ArcGIS version 10.3 was used for doing the Moran I analysis.

### Spatial scan statistical analysis

Spatial scan statistical analysis was employed to identify the geographical locations of statistically significant spatial clusters of health coverage among women aged 15–49 years using Kuldorff’s SaTScan version 9.6 software. Spatial scan statistic used a scanning window that moves across the study area. Scan statistics did scan gradually across the space to identify the number of observed and expected observations inside the window at each location. The scanning window with the maximum likelihood was the most likely high performing clusters, and a *p*-value was assigned to this cluster [24]. The maximum cluster size was set at 50% of the population at risk. Women without health insurance coverage taken as controls and those covered with health insurance were taken as cases represented by a 0/1 variable to fit the Bernoulli model. The number of cases in each location had Bernoulli distribution and the model required data with or without health insurance coverage. A Likelihood ratio test statistic was used to determine whether the number of observed insured cases within the potential cluster was significantly higher than the expected or not. Primary and secondary clusters were identified using *p*-values and likelihood ratio tests based on the 999 Monte Carlo replications [24].

## Results

### Characteristics of study population

A total of 15,683 reproductive age group women were interviewed. The majority (78%) of the respondents were rural residents. Regarding the level of education, 47.81% of them did not have any formal education. The household wealth of 34.7% of the study subjects were in the two poor wealth quintiles, 18.99% were in the middle and 46.31% were in the two upper wealth quintiles (Table 2).

**Table 2:** Sociodemographic characteristics of respondents in Ethiopia from January 18 to June 27, 2016 (*N* = 15,683)

Variables	Weighted frequency	Percent
**Residence**		
Urban	3476	22.16
Rural	12,207	77.84
**Age**		
15–19	3381	21.56
20–24	2761	17.61
25–29	2957	18.85
30–34	2345	14.95
35–39	1932	12.32
40–44	1290	8.22
45–49	1017	6.48
**Occupation**		
Not working	7819	49.86
Professional	381	2.43
Clerical	144	0.92
Sales	2354	15.01
Agricultural	3263	20.81
Services	542	3.46
Skilled manual	602	3.84
Unskilled manual	208	1.32
Others	370	2.36
**Wealth quintile**		
Poorest	2633	16.79
Poorer	2809	17.91
Middle	2978	18.99
Richer	3100	19.76
Richest	4163	26.55
**Level of education**		
No education	7498	47.81
Primary	5490	35.01
Secondary	1818	11.59
Higher	877	5.59
**Region**		
Tigray	1129	7.20
Afar	128	0.82
Amhara	3714	23.68
Oromia	5701	36.35
Somali	460	2.93
Benishangul	160	1.02
SNNPR	3288	20.97
Gambella	44	0.28
Harari	39	0.25
Addis Ababa	930	5.93
Dire Dawa	90	0.58
**Marital Status**		
Never married	4037	25.74
Married/living together	10,223	65.19
Divorced/separated/widowed	1423	9.08

### Spatial distribution of health insurance coverage

**Fig. 1:**
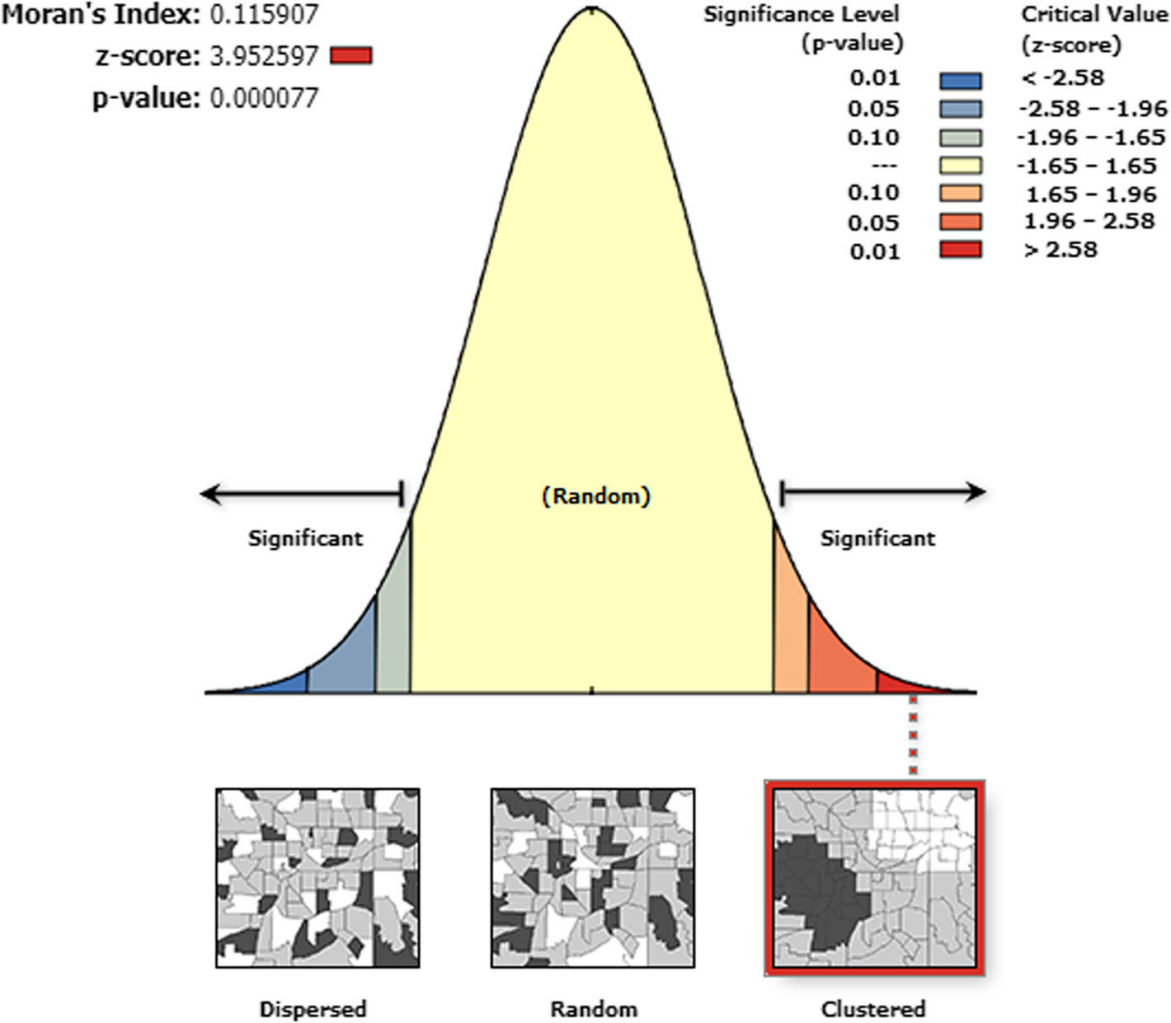
Spatial autocorrelation analysis of health insurance coverage in Ethiopia, 2016

This study revealed that the spatial distribution of health insurance coverage was found to be spatially clustered in Ethiopia with Global Moran’s I 0.115 (*p* < 0.001). Cluster of high rates in health insurance coverage was observed over the study area. The outputs were automatically generated keys on the right and left sides of each panel. Given the z-score of 3.9 indicated that there is less than 1% likelihood that this clustered pattern could be the result of chance. The bright red and blue colours to the end tails indicates an increased significance level (Fig. 1).

**Fig. 2:**
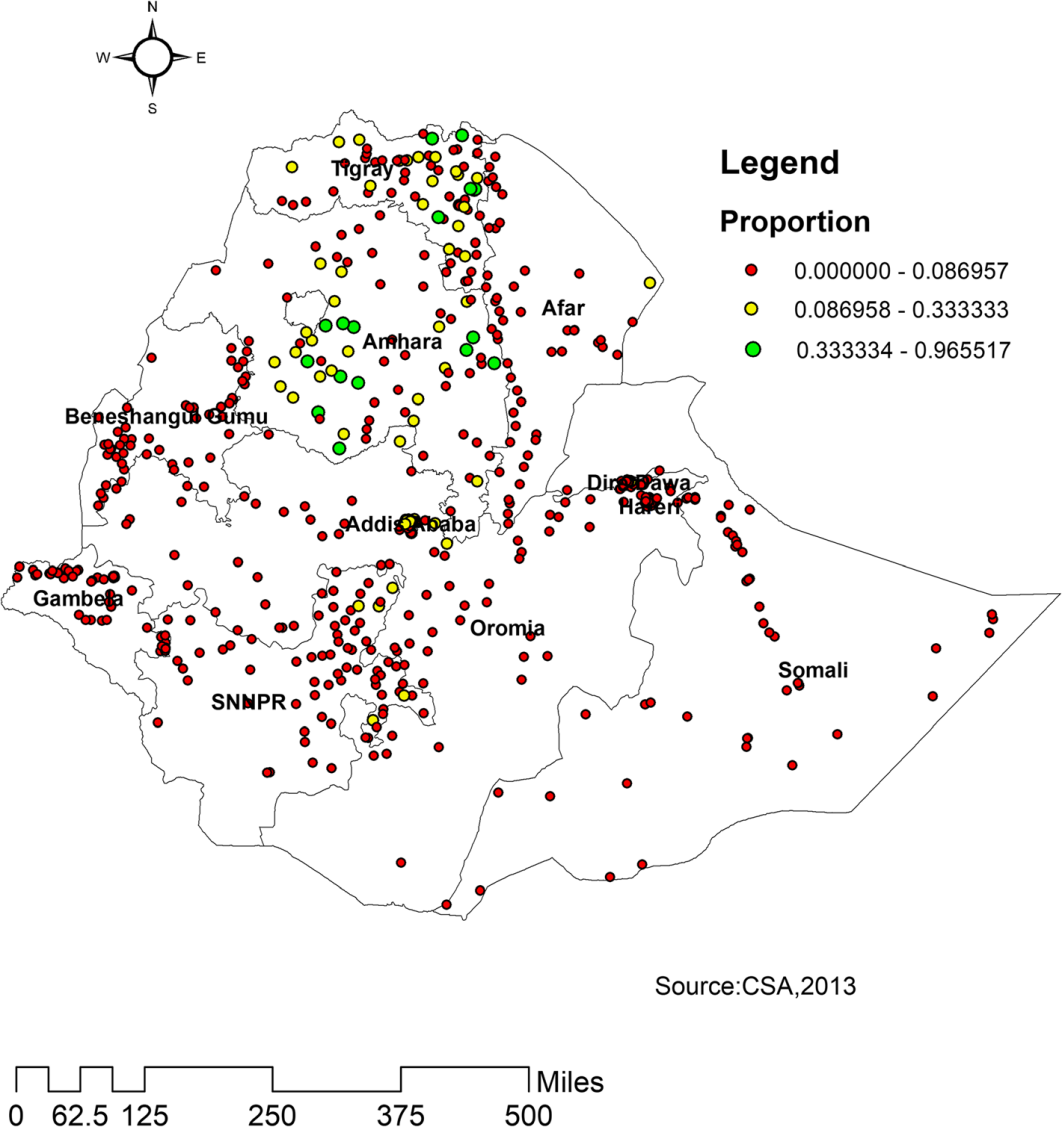
Spatial distribution of health insurance coverage across regions in Ethiopia, 2016

Spatial clustering of health insurance coverage was found at regional levels. Of a total of 15,683 women interviewed in 2016, only 830 (5.29%) had health insurance coverage. The highest health insurance coverage was spatially clustered in Amhara and Tigray, while Harari, Diredawa, Gambella, Oromia, Benishangul and Somali regions had the lowest health insurance coverage (Fig. 2).

### Spatial SaTScan analysis of health insurance coverage (Bernoulli based model)

Most likely (primary clusters) and secondary clusters of health insurance coverage were identified. In EDHS 2016, spatial scan statistics identified a total of high and modest performing spatial clusters of health insurance coverage. Of these, 153 clusters were high performing clusters (LLR = 268.09, RR = 6.04, *P* < 0.001) and 18 clusters were lowest performing clusters (LLR = 18.18, RR = 2.44, *P* < 0. 001. The highest performing clusters of health insurance coverage were detected in Amhara, Tigray, southeast Benishangul and western part of Afar regions (Table 3).

**Table 3:** Significant spatial clusters with high rate health insurance coverage among women in Ethiopia, 2016

Cluster	Enumeration area (cluster)identified	Coordinate (radius)	Population	Case	RR	LLR	*P* value
1	640,638,312,327,152,322,163, 292, 279, 80, 628, 199, 158,612,512,296,169,132,504,258,425,73,431,188,340, 516,456,167,551,382,253,542,52,583,66,627,259,403,361,429,579,98,602,181,24,575,38,156,255,584,415,636,300, 268,528,545,541,538,120,392,597,136,401, 400, 424, 109, 78 386,548,590,591,81,143,515,355,3, 478, 160, 615, 449, 498, 430, 84, 97, 351, 481, 237, 94, 550, 442, 176,604,200, 375, 206, 45, 605, 455, 128, 79, 384, 461, 220, 474, 533, 129, 354, 226, 246, 421, 479, 249, 488, 623,482,89,559,460,616,496,99, 410, 511, 298, 341, 10, 229,531,598,617,36,130,196,332, 404, 344, 494, 172, 189,150,350,241, 267, 18, 127, 611, 218, 183, 571, 345, 235,389,413	(12.673719 N, 37.510687 E)/295.08 km	3800	439	6.04	268.09	< 0.001
2	303, 90, 402, 40, 287, 211, 560, 509, 330, 428, 155, 247, 19, 639,464, 15, 153, 293	(9.041754 N, 38.986283 E)/24.36 km	602	60	2.44	18.18	< 0.001

**Fig. 3:**
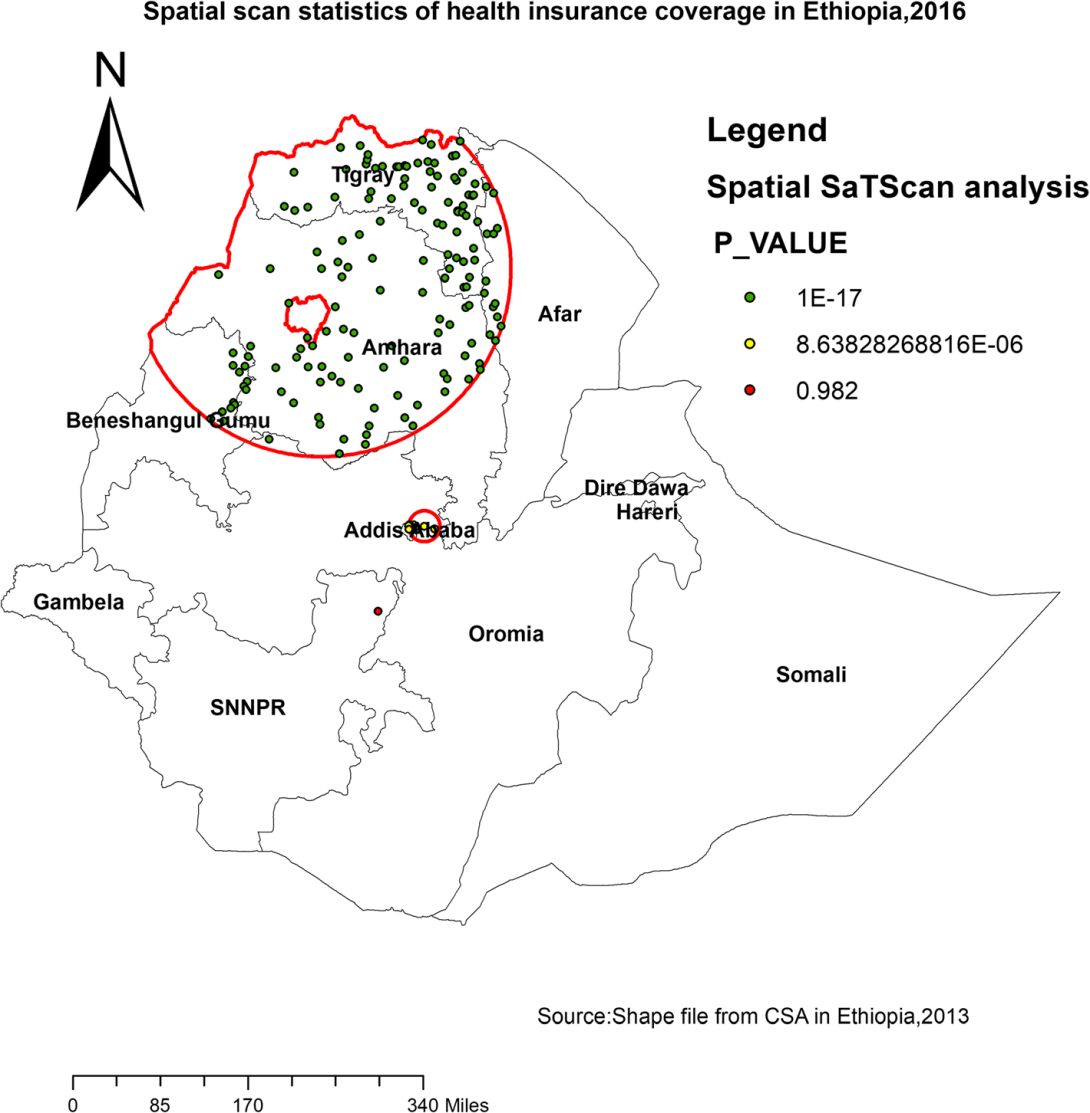
Primary and secondary clusters of health insurance coverage among women across regions in Ethiopia, 2016

The bright red colors (rings) indicates that the most statistically significant spatial windows of health insurance coverage. There was high insurance coverage within the cluster than outside the cluster (Fig. 3).

### Factors associated with health insurance coverage

In the bivariable mixed-effect logistic regression model, maternal age, family wealth quintile, maternal occupation, residence, maternal educational level, family size, frequency of reading newspaper, frequency of listening to radio and frequency of watching television were associated with health insurance coverage (*p* < 0.2). However, in the multivariable mixed-effect logistic regression analysis maternal age, family wealth quintile, maternal occupation, maternal education, family size and frequency of reading newspapers were significantly associated with health insurance coverage (*p* ≤ 0.05).

Women with age of 40–44 and 45–49 years had 2 and 1.6-times higher odds of being covered by health insurance compared to those aged 15–19 years respectively [(AOR = 2.14, 95% CI = 1.37–3.35), (AOR = 1.61, 95% CI = 1.00–2.63)].

The probability of being covered by health insurance increased with increased wealth index. The richest wealth quintiles were 3.43 times more likely to be covered than those in the lowest wealth quintiles. The richer wealth quintiles were 3.29 times more likely to be covered than those in the lowest wealth quintiles. The middle wealth quintiles were 2.44 times more likely to be covered than those in the lowest wealth quintiles.

Employed women were more likely to be covered with health insurance as compared with the unemployed once. Mothers who completed secondary and higher education were 1.76 and 2.62 times more likely to be covered by health insurance than those with no education respectively. Mothers who read a newspaper at least once a week were 1.78 times more likely to be covered by health insurance than those who didn’t read newspaper [AOR = 1.78; 95% CI; 1.18–2.68].

Mothers who had more than 5 household members were 1.25 times more likely to be covered with health insurance coverage than those who had 5 and less household members (AOR = 1.25,95% CI = 1.01,1.55) (Table 4).

**Table 4:** Bi-variable and multivariable mixed-effect logistic regression analysis of health insurance coverage among women in Ethiopia, 2016 (*N* = 15,683)

Variables	Health Insurance		Odds Ratio (95% CI)	
	Yes	No	COR	AOR
**Residence**				
Urban	185	3291	1	1
Rural	645	11,562	0.35 (0.22,0.58)	0.95 (0.48,1.84)
**Age**				
15–19	186	3195	1	1
20–24	114	2648	0.91 (0.66,1.25)	0.79 (0.55,1.12)
25–29	112	2845	1.12 (0.82,1.52)	0.98 (0.67,1.44)
30–34	116	2229	1.31 (0.95,1.80)	1.31 (0.86,1.97)
35–39	124	1,8 09	1.41 (1.03,1.93)	1.43 (0.95,2.17)
40–44	99	1191	2.13 (1.52,3.01)	2.14 (1.37,3.35)*
45–49	80	937	1.46 (0.98,2.16)	1.62 (0.99,2.64)
**Occupation**				
Not working	248	7571	1	1
Professional	33	348	4.66 (3.12,6.97)	2.41 (1.50,3.87)*
Clerical	19	125	7.97 (4.82,13.2)	4.33 (2.50,7.49)*
Sales	85	2269	1.01 (0.73,1.40)	1.02 (0.73,1.42)
Agricultural	350	2913	1.74 (1.31,2.31)	1.76 (1.31,2.37)*
Services	12	530	1.17 (0.66,2.09)	1.12 (0.62,2.01)
Skilled manual	50	551	2.09 (1.38,3.16)	2.08 (1.36,3.16)*
Unskilled manual	19	189	1.21 (0.64,2.25)	1.37 (0.72,2.60)
Others	15	356	2.19 (1.28,3.75)	1.87 (1.09,3.24)*
**Wealth status**				
Poorest	61	2572	1	1
Poorer	118	2691	1.87 (1.21,2.89)	1.77 (1.13,2.75)*
Middle	193	2786	2.62 (1.70,4.05)	2.45 (1.57,3.82)*
Richer	239	2860	3.83 (2.47,5.95)	3.29 (2.08,5.21)*
Richest	219	3944	4.94 (3.10,7.86)	3.43 (1.95,6.01)*
**Level of education**				
No education	389	7109	1	1
Primary	260	5231	1.03 (0.81,1.31)	1.18 (0.88,1.57)
Secondary	114	1703	1.67 (1.23,2.26)	1.77 (1.21,2.58)*
Higher	67	810	4.08 (2.90,5.76)	2.62 (1.63,4.23)*
**Marital Status**				
Single	223	3814	1	1
Married	511	9712	1.13 (0.91,1.41)	1.03 (0.76,1.39)
Divorced/widowed	96	1327	0.95 (0.68,1.32)	0.93 (0.62,1.38)
**Family size**				
< 5	293	5329	1	1
≥ 5	537	9524	1.26 (1.03,1.53)	1.25 (1.01,1.55)*
**Reading newspaper**				
Not at all	696	12,852	1	1
Less than once a week	94	1422	1.83 (1.40,2.38)	1.34 (0.99,1.81)
At least once a week	40	579	2.78 (1.93,4.01)	1.78 (1.18,2.68)*
**Listening to radio**				
Not at all	536	9949	1	1
Less than once a week	162	2455	1.12 (0.94,1.56)	0.89 (0.67,1.17)
At least once a week	132	2449	1.43 (1.10,1.86)	0.91 (0.68,1.22)
**Watching television**				
Not at all	570	10,725	1	1
Less than once a week	137	1766	1.27 (0.93,1.73)	0.98 (0.70,1.37)
At least once a week	123	2362	1.78 (1.29,2.46)	0.96 (0.65, 1.41)

*P ≤ 0.05

## Discussion

This study amid to identify the spatial distribution of health insurance coverage and factors associated with it in Ethiopia. In this study, the distribution of health insurance coverage among women varied in the country. The Global Moran’s I value 0.11 (*p* < 0.001) indicated that there was a significant clustering of health insurance coverage in the study area.

The highest health insurance coverage was reported in Amhara, Tigray, southeast Benishangul and western part of Afar regions, whereas comparatively modest utilization was reported in Addis Ababa. This could be due to these regions are relatively urban in which health facilities are more accessible and women are more aware of health insurance. Another possible reason for this difference could be due to the difference in access to tertiary level care and premium payment methods across regions. In Amhara and Tigray, CBHI enrolees may visit any public hospital within the region. This increase the accessibility of health insurance service [25].

This study revealed that maternal age as a predictor of health insurance coverage. Relatively older women were more likely to have health insurance coverage as compared to younger women. Similar studies have also reported a direct relationship between maternal age and health insurance coverage [13, 15, 17]. This could be explained by as maternal age increases, their health status deteriorates. As a result, the need for healthcare utilization increases just to refrain from severe financial catastrophe associated with health problems. Moreover, since older women had higher chance of being employed, they didn’t have financial problems to be a member of health insurance compared to younger once.

Even though, health insurance schemes designed to help ease the financial burden on the poorest households, the finding of this study revealed that the odds of health insurance coverage was higher among women in the richest wealth quintile. This finding is consistent with those from other settings elsewhere [13, 15–17, 26]. This could be households with high wealth quantiles often have more income to afford health insurance premium compared to the poor once.

Consistent with previous studies [13, 15–17], the current study showed that the likelihood of being covered by health insurance was highest among educated women as compared with women with no education. The possible explanation could be educated women have more knowledge about the advantages of health insurance and make informed choices, engage themselves in different knowledge enhancement activities like reading materials, give more concern for their health and insure themselves against the unexpected out-of-pocket payments.

Being a worker was a significant predictor of health insurance coverage among women in this study. This finding is consistent with similar studies done in Africa [15, 26]. The possible reason could be employed women are economically empowered to pay premium contributions than the unemployed once.

Reading newspapers one significant predictor of health insurance coverage. This finding is consistent with the study done in Kenya [26]. This could be due information related health insurance and help mothers to make informed choice regarding health insurance.

Family size is significantly associated with health insurance coverage. This result is consistent with a study conducted in China [27], where mothers who had more than 5 household members were more likely to be covered by health insurance than those who have a small number of household members. The possible justification could be the out-of-pocket payments would be higher for the large household group.

One of the strengths of this study was using large population-based data with a large sample size, which is representative at national and regional levels, so it can be generalized to all women in reproductive age group in Ethiopia. Furthermore, the combined use ArcGIS and Sat Scan statistical tests helped to detect similar and statistically significant area with low health insurance coverage (hot spot area). This study is not free from limitations. First, the location of data values was shifted 1-2kms for urban and 5kms for rural areas for data confidentiality issues, thus, this was the challenge to know the exact cases’ location. Since EDHS data was secondary data some potentially important predictors were not available like source of primary care, self-reported illness and satisfaction with cost of care. Recall bias might be the other limitation for this study as EDHS was a questionnaire-based survey and relied on the memory of the respondents. Despite the above limitations, this study can provide valuable information about the spatial disparity of health insurance coverage and identify hotspot areas to make local interventions through visualization and cluster analysis.

## Conclusion

This study identified spatial clusters of health insurance coverage in Diredawa, Harari, Gambella, Benishangul and western SNNPRs with low health insurance coverage rate and Amhara, Tigray and western part of Afar with the highest health insurance coverage rate. Being older, richest wealth quantile, having more than 5 family members, educated, having occupation and reading newspapers were predictors that increased the odds of health insurance coverage. There is a concern of inequity in health insurance coverage in Ethiopia. Ministry of health, health bureaus and partners better to give priority for clusters with low health insurance coverage in order to strengthen health system financing and act on factors triggering low coverage health insurance.

## Data Availability

All necessary information’s were included with in the manuscript.

## Abbreviations

AOR: Adjusted Odds Ratio
CBHI: Community Based Health Insurance
COR: Crude odds ratio
CSA: Central Statistics Agency
EDHS: Ethiopian Demographic and Health Survey
SHI: Social Health Insurance
SNNPR: Southern Nation and Nationality and Peoples Region
WHO: World Health Organization
